# Rhinophyma: A Report of Two Patients Managed With Electrosurgery

**DOI:** 10.7759/cureus.112465

**Published:** 2026-07-11

**Authors:** Houda El Abbade, Hanane Baybay, Riham Alheyasat, Sara Elloudi, FatimaZahra Mernissi

**Affiliations:** 1 Dermatology, Hassan II University Hospital, Sidi Mohamed Ben Abdellah University, Fez, MAR

**Keywords:** aesthetic outcomes, deformity, electrosurgery, nasal, rhinophyma, sebaceous glands

## Abstract

Rhinophyma is a chronic cutaneous condition secondary to rosacea that primarily affects the nose, leading to a bulbous and enlarged appearance. We report two cases of rhinophyma in 65- and 76-year-old men with long-standing rosacea evolving for more than 10 years. Both patients presented with progressive nasal enlargement, erythema, and nodular hypertrophy that had worsened over the previous five to seven years, leading to significant cosmetic and psychosocial impairment. The diagnosis was clinical. Surgical treatment was performed in both cases, including electrosurgical excision in one patient, allowing effective removal of hypertrophic tissue with good hemostasis and nasal remodeling. Postoperative care combined light-emitting diode photobiomodulation with adapted wound dressings and topical healing agents according to the stages of wound healing. Outcomes were favorable, with marked aesthetic improvement, reduction of nasal hypertrophy, and improved quality of life in both patients. Rhinophyma may significantly impair quality of life because of its progressive and disfiguring nature. Early management of rosacea with appropriate medical treatment is essential to limit progression; however, advanced hypertrophic forms may require therapeutic escalation to surgical approaches such as electrosurgery. In such cases, electrosurgical remodeling associated with appropriate postoperative wound care can provide favorable functional and aesthetic outcomes.

## Introduction

Rhinophyma, derived from the Greek words “rhis” (nose) and “phyma” (growth), refers to the enlarged, bulbous, and reddened appearance of the nose, typically resulting from granulomatous infiltration, often as a consequence of untreated rosacea [1].

Rhinophyma is an uncommon and late manifestation of rosacea, most frequently affecting individuals in their 40s to 60s, and is rarely observed before the age of 30. Epidemiological data suggest a marked male predominance and a higher prevalence among Caucasian populations, particularly individuals of Northern European descent [2]. The onset is subtle, and the progression is chronic [1,2]. The fundamental problem the patients present with is cosmetic deformity. Clinical evolution is slow and often neglected by the patient.

Although the exact pathophysiology remains incompletely understood, rhinophyma is thought to result from chronic inflammation leading to sebaceous gland hyperplasia, connective tissue overgrowth, and vascular proliferation. Therapeutic management is challenging, particularly in advanced stages, where surgical and laser-based approaches are often required.

## Case presentation

We report two cases of rhinophyma in male patients aged 65 and 76 years who presented with progressive nasal enlargement, erythema, and acneiform lesions occurring in the context of long-standing rosacea. In both patients, rosacea had been present for more than 10 years, with a marked worsening during the previous five to seven years.

Both patients presented to our department with a gradual increase in nasal volume associated with irregular skin texture and the appearance of multiple erythematous nodules. The progression was slow and progressive, evolving over approximately five years in the first patient (Figure 1) and seven years in the second (Figure 2). During this period, the nasal tissue progressively became bulbous, thickened, and hypertrophic (Figure 2A).

**Figure 1:**
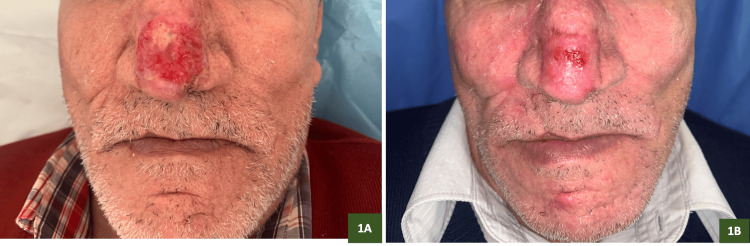
Clinical appearance for the first patient. (A) Post-excision aspect showing a well-demarcated ulceration with irregular borders, a partially clean surface, and central fibrinous areas. (B) After six sessions of light-emitting diode photobiomodulation, near-complete re-epithelialization of the ulcerated area can be noted.

**Figure 2:**
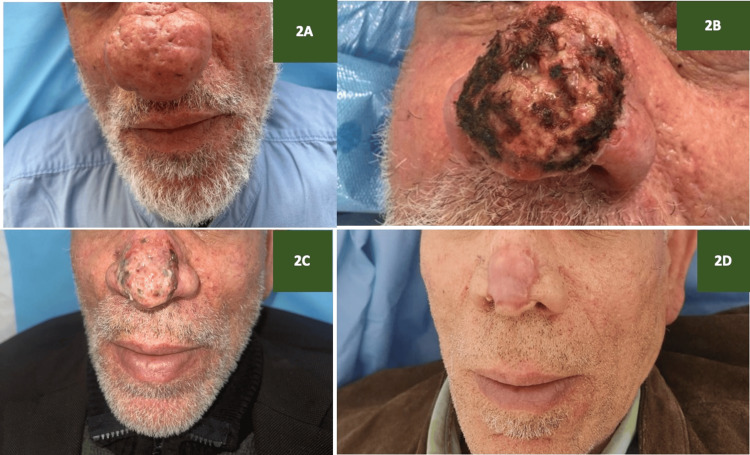
Clinical appearance of the second patient. (A) Clinical presentation of rhinophyma showing bulbous nasal enlargement with irregular surface and erythematous nodules. (B) Post-excision stage showing extensive ulceration covered with hemorrhagic crusts involving most of the nasal surface. (C) Well-defined ulceration with a fibrinous base and peripheral meliceric (honey-colored) crusts. (D) Final outcome showing an irregularly oval scar with resolution of the bulbous nasal deformity.

Neither patient reported pain or functional impairment; however, the nasal deformity had a considerable psychosocial impact, particularly affecting self-esteem and social interactions. The diagnosis of rhinophyma was established based on clinical examination.

The first patient underwent surgical treatment performed by the maxillofacial surgery team (Figure 1A). The second patient was treated in our dermatology department by surgical excision of the hypertrophic nasal tissue using electrosurgery under local anesthesia with xylocaine (lidocaine) (Figure 2B).

Electrosurgery consists of the application of high-frequency electrical current to biological tissues to achieve cutting, coagulation, and tissue vaporization. This technique is particularly suitable for the management of rhinophyma because it allows precise removal of hypertrophic and fibrotic tissue while ensuring effective hemostasis and facilitating nasal contour remodeling.

Postoperative wound care was provided for both patients and included multiple sessions of light-emitting diode (LED) photobiomodulation combined with stage-appropriate topical treatments according to the different phases of wound healing. Initial management during the hemorrhagic phase consisted of alginate dressings, followed by hydrocolloid dressings during the fibrinous phase, along with the application of reparative topical creams until complete re-epithelialization was achieved.

Postoperative outcomes were favorable in both cases. Patient 1 demonstrated a marked improvement in nasal morphology with resolution of the nodular deformity (Figure 1B). Patient 2 showed a significant reduction in nasal volume with satisfactory cosmetic results and a notable improvement in quality of life (Figures 2C, 2D).

## Discussion

Rhinophyma represents the most severe form of phymatous rosacea and is characterized by progressive hypertrophy of sebaceous glands, connective tissue overgrowth, and chronic inflammation of the nasal skin. It predominantly affects older men, typically between the fifth and seventh decades of life, which is consistent with the demographic characteristics of our patients [1].

The pathophysiology of rhinophyma remains incompletely understood but is considered multifactorial. Chronic inflammation, vascular dysregulation, sebaceous gland hyperplasia, and dermal fibrosis are key mechanisms involved. Additional contributing factors, such as *Demodex folliculorum* colonization and innate immune dysfunction, have also been described [1]. The long-standing history of rosacea observed in both of our patients, exceeding 10 years with progressive worsening, aligns with the natural history reported in previous studies, where rhinophyma develops as a late-stage complication [1-3].

Clinically, rhinophyma presents with progressive nasal enlargement, irregular skin thickening, and nodular deformities, often associated with erythema and telangiectasia. These features were clearly observed in our cases. The slow progression over several years, leading to a bulbous and hypertrophic nasal appearance, is consistent with findings reported in the literature, including case-based analyses describing the clinical spectrum of phyma variants [4].

The diagnosis of rhinophyma is primarily clinical; however, histopathological examination may be warranted in atypical cases to exclude malignancies such as basal cell carcinoma. Although a biopsy was not performed in our patients, the clinical presentation was typical, allowing for a confident diagnosis [1].

Management of rhinophyma depends largely on disease severity. Medical therapies, including oral antibiotics and isotretinoin, may be beneficial in early inflammatory stages but are generally ineffective in advanced phymatous forms. As emphasized in systematic reviews and recent studies, surgical intervention remains the gold standard for advanced disease [1,2].

Several surgical modalities have been described, including scalpel excision, dermabrasion, carbon dioxide laser, and electrosurgery. Each technique offers specific advantages and limitations. Electrosurgery, as used in our second patient, allows precise removal of hypertrophic tissue with simultaneous hemostasis, thereby facilitating contouring of the nasal architecture. This approach has been specifically highlighted in studies focusing on high-frequency electrosurgery, demonstrating favorable cosmetic outcomes and reduced intraoperative bleeding [4,5].

In contrast, our first patient underwent management by maxillofacial surgery, highlighting the multidisciplinary nature of rhinophyma treatment, especially in advanced or extensive cases. Surgical management remains highly effective in restoring both function and aesthetic appearance, as also reported in several case reports and reviews [6,7].

Postoperative wound care plays a crucial role in optimizing surgical outcomes. In our cases, healing was supported by adapted local wound care combined with LED photobiomodulation. Previous studies have suggested that photobiomodulation may enhance tissue repair, reduce postoperative inflammation, and improve wound healing quality, particularly in extensive surgical defects [8].

Both of our patients demonstrated favorable outcomes, with a significant reduction in nasal deformity and improvement in quality of life. These results are consistent with the literature, which reports high patient satisfaction and good cosmetic outcomes following surgical management of rhinophyma [7].

## Conclusions

Rhinophyma is an advanced manifestation of rosacea leading to progressive nasal deformity and significant psychosocial impact. Our cases highlight the typical long-standing evolution and the need for surgical management in advanced stages. Electrosurgery provides an effective and precise approach, allowing adequate tissue debulking with good hemostasis and satisfactory cosmetic outcomes. Early diagnosis and appropriate treatment are essential to prevent progression and improve patients’ quality of life.
